# Synthesis of ^99m^Tc-labeled polyaspartic acid/silica nanoassembly as a potential probe for bone imaging

**DOI:** 10.1186/s13065-025-01508-z

**Published:** 2025-05-24

**Authors:** Noha A. Bayoumi, Marwa E. Sayyed, Wael M. Darwish

**Affiliations:** 1 Department of Radiolabeled Compounds, Hot Labs Centre, Egyptian Atomic Energy Authority (EAEA), 13759 Cairo, Egypt; 2 Department of Polymers and Pigments, National Research Centre, Elbohooth Street, Dokki12622, Giza, Egypt

**Keywords:** Mesoporous silica nanoparticles, Polyaspartic acid – Technetium-99m, Bone imaging

## Abstract

**Purpose:**

Due to the efficient bone targeting of mesoporous silica nanoparticles (MSNs) and polyaspartic acid (PASP), ^99m^Tc- labeled polyaspartic acid coated mesoporous silica nanoparticles (PASP-mSiO_2_-DTPA-^99m^Tc) are proposed as a potential probe for bone imaging.

**Methods:**

Polyaspartic acid-conjugated silica nanoparticles (PASP-mSiO_2_) were synthesized using aqueous carbodiimide chemistry and characterized by ATR-FTR, FE-SEM, EDX, TEM, TGA and XRD. Radiolabeling of the produced nanoassembly with ^99m^Tc was carried out via a simple DTPA chelation procedure. Aqueous dispersion of the radiolabeled nanoparticles was intravenously injected into normal mice and the bone targeting efficiency was evaluated.

**Results:**

The PASP-mSiO_2_ nanoassembly was efficiently synthesized and radiolabeled with ^99m^Tc with a high radiochemical yield (92 ± 0.5%) and sufficient in vitro stability in PBS and FBS for up to 24 h. In vivo biodistribution studies revealed a significant enhancement of radioactivity bone uptake after intravenous injection of PASP-mSiO_2_-DTPA-^99m^Tc compared to radiolabeled uncoated MSNs (mSiO_2_-DTPA-^99m^Tc), (13 ± 0.6% IA/gram and 5.4 ± 0.4, respectively).

**Conclusion:**

PASP endowed MSNs with enhanced biocompatibility and highly selective bone targeting. Therefore, the proposed PASP-mSiO_2_-DTPA-^99m^Tc nanoassembly has immense potential in the field of bone- imaging via single photon emitting computed tomography (SPECT).

**Graphical Abstract:**

**Figure:**
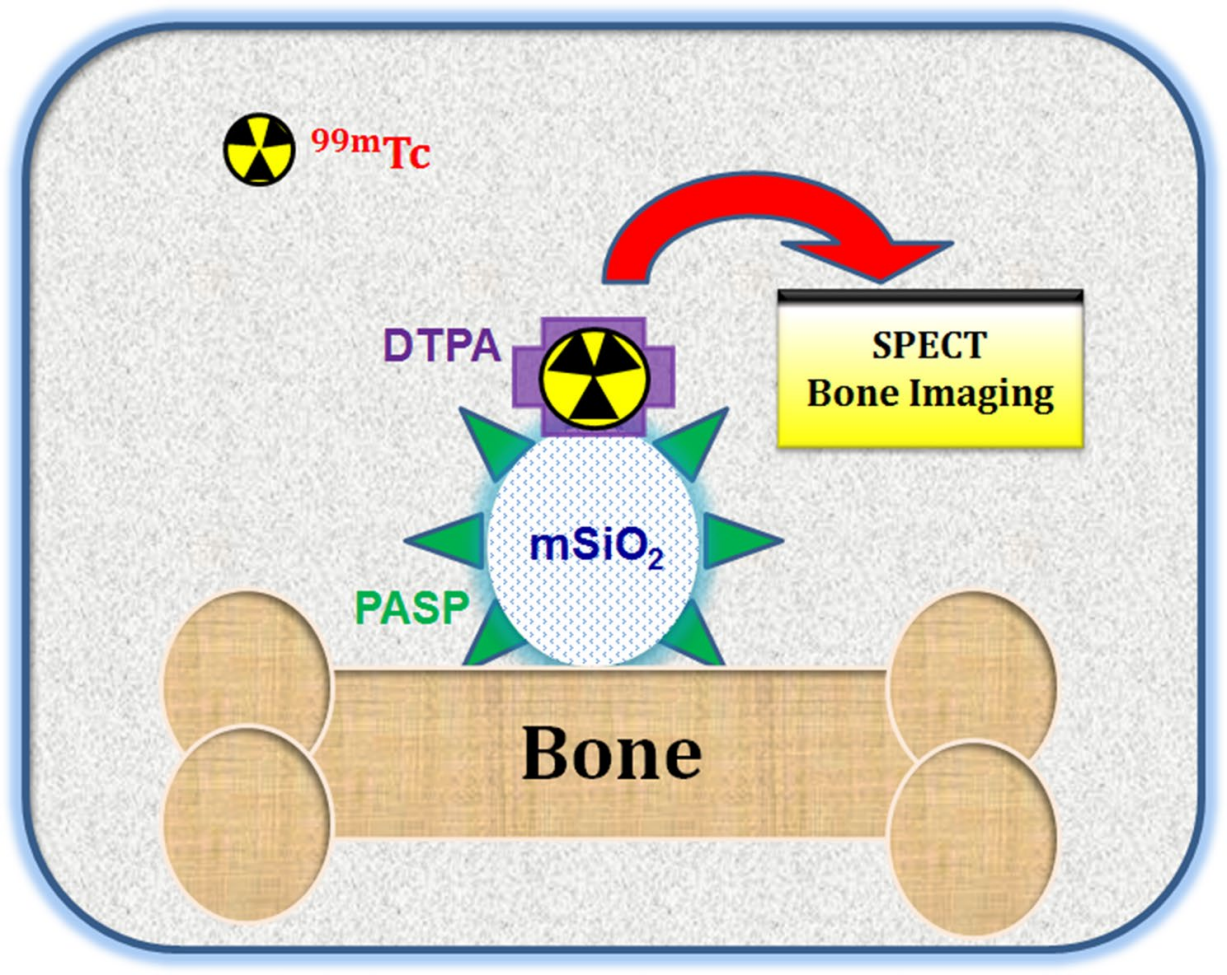
.

**Supplementary Information:**

The online version contains supplementary material available at 10.1186/s13065-025-01508-z.

## Introduction

Technetium-99m (^99m^Tc) is currently the most widely used radiotracer for diagnostic imaging with single photon emission computed tomography (SPECT). The low-cost production and short half-life (t_1/2_ = 6 h) of this isotope have motivated many researchers to develop advanced SPECT bone imaging probes based on ^99m^Tc [1]. Although, ^99m^Tc radiopharmaceuticals are clinically applied for whole-body bone scans such as ^99m^Tc- methylene bisphosphonate (^99m^Tc-MDP) however, false-negative bone SPECT imaging has been reported in patients with previous bisphosphonate (BP) treatment [2]. To address this issue, recent investigations have focused on designing radiopharmaceuticals that target sites other than the bisphosphonate binding site in the bone. Nanotechnology has the potential to significantly improve the SPECT technique. ^99m^Tc-based radiolabeling of nanoparticles has opened new avenues for the production of sensitive clinical imaging SPECT probes [3]. Mesoporous silica nanoparticles (MSNs) have been extensively explored in bone-targeted nanomedicine as therapeutic or diagnostic agents capable of exclusively reaching bone diseases [4]. MSNs are widely used for the efficient treatment of complex bone diseases such as osteoporosis [5] and bone infection [6] in addition to their use in bone tissue regeneration [7]. Functionalization of MSNs, either with bone-targeting molecules or biopolymers, has been intensively utilized to construct nanoassemblies with enhanced bone-targeting [8, 9]. Polyaspartic acid (PASP) is a water soluble polymer with a linear polyamide backbone structure that enables rapid and complete biodegradation [10]. PASP is proposed for this study due to its high affinity for hydroxyapatite making it effective as a bone targeting moiety to deliver drugs to bone [11, 12]. In vitro and in vivo studies have revealed that a short peptide sequence of the aspartic acid interacts exclusively with bone and teeth [13]. In the last decade, the aspartic acid peptide sequence has been frequently used to target small drugs to bone tissue [14]. For example, PASP has been successfully used to promote bone accumulation of small molecular weight agents, such as radiogallium-labeled bone imaging agent [15].

In this work, the attractive bone-targeting features of polyaspartc acid (PASP) and mesoporous silica nanoparticles (MSNs) have prompted us to develop a novel ^99m^Tc -labeled nanoassembly based on PASP covalently anchored onto the surface of amino-functionalized mesoporous silica as a potential probe for bone-targeting drug delivery applications. The proposed imaging nanoprobe is featured with a safe biological fate due to the biodegradability of PASP and the biocompatibility of MSNs.

## Materials and methods

### Materials

Sterile Milli-Q ultrapure water with a resistivity of 18.2 MΩ·cm at 25 °C was used throughout the work. N-hydroxysuccinimide (NHS), N-(3-dimethylaminopropyl)-N′-ethylcarbodiimide hydrochloride (EDC) 98%, N, N′-dicyclohexylcarbodiimide (DCC), diethylenetriamine-pentaacetic acid (DTPA), cetyltrimethylammonium bromide (CTAB) were products of Sigma-Aldrich Co., Germany. Maleic anhydride, tetraethyl orthosilicate (TEOS), 3-aminopropyltriethoxysilane (APTES), pyridine and acetic anhydride were products of ACROS Organics, Belgium. Aqueous ammonia (33%) was procured locally from ADWK, Egypt. Dimethylformamide (DMF) and dimethylsulfoxide (DMSO) were of analytical grade and dehydrated by fractional distillation under reduced pressure.Technetium-99m (^99m^Tc) was eluted in the form of ^99m^TcO_4_¯ from the ^99^Mo/^99m^Tc generator, at the Radioisotope Production Facility, EAEA, Egypt.

### Instruments

UV-Vis absorption measurements were recorded using a computerized recording on a Cary 300 spectrophotometer, from Agilent Technologies. ATR-FTIR was measured using a Bruker VERTEX 80 (Germany) combined with Platinum Diamond ATR, with a range of 4000–400 cm^-1^ with a resolution of 4 cm^-1^, and a refractive index of 2.4. The morphological structure was studied using field emission scanning electron microscopy (FE-SEM) using with a JEOL instrument, JXA–840 A. Energy dispersive X-ray spectroscopy (EDS) was done on an INCAx–Sight from Oxford Instruments. Transmission electron microscope (TEM) images were recorded on a JEM-2100, Jeol electron microscope. Dynamic light scattering (DLS) instrument from PSS, Santa Barbara, CA, USA, using the 632 nm line of a He-Ne laser as the incident light with an angle of 90^o^ and Zeta potential with an external angle of 18.9^o^. Thermal gravimetric analysis (TGA) was performed under nitrogen using a Perkin Elmer Thermogravimetric Analyzer TGA7, USA (RT to 600 ºC) with a heating rate of 10 °C min^-1^. X-ray diffraction (XRD) data were collected on a PANalytical EMPYREAN diffractometer from Holland with an operating voltage of 45 kV using CuKα as a radiation source. Diffraction patterns were recorded in the angular range of 10–80 with a step width of 0.02 s. Proton Nuclear Magnetic Resonance (^1^H-NMR) spectra were recorded on a Bruker 400 MHz NMR using d_6_-chloroform as a solvent. For γ counting, a γ-Scintillation counter (Scaler Ratemeter SR7, Nuclear enterprises LTD, USA fitted with a well type NaI (TI) crystal) was utilized.

### Synthesis

#### Synthesis of mSiO 2-NH2 Nanoparticles

Amino-functionalized mesoporous silica nanoparticles were prepared using a one-pot co-condensation method as previously described by Wada et al. [16] with slight modifications. In brief, a mixture of CTAB (0.7 g, 1.9 mmol) in Milli-Q ultrapure water (350 mL) and 3 ml of 2 M NaOH were stirred at 75 °C for 15 min. TEOS (3.5 mL, 18 mmol) and APTES (0.43 mL, 2.04 mmol) were then added and the mixture was stirred for 3 h. The white solid was isolated by filtration under reduced pressure using a glass frit and subsequently washed with water and ethyl alcohol. After drying at 60 °C, CTAB was removed by soxhlet extraction using a solution of isopropyl alcohol (100 mL) and HCl 37% (5 mL).

#### Synthesis of mSiO2-DTPA nanoparticles

Firstly DTPA dianhydride (DTPA-DA) was prepared as previously described [17]. For the preparation of *m*SiO_2_-DTPA, a mixture of silica (300 mg), DTPA-DA (357 mg, 1 mmol), EDC.HCl (230 mg, 1.2 mmol), NHS (115 mg, 1 mmol) and 15 mL of anhydrous DMF was heated at 50 °C in the dark under static argon for 3 h. After cooling to room temperature, the yellowish product was separated by centrifugation (8000 rpm, 10 min). The solid was washed with a mixture of methanol, DI water and DMF (10:3:0.5). Finally the solid was thoroughly washed with acetone and dried under reduced pressure. The solid was stored in the dark under argon at 4 °C.

#### Synthesis of polyaspartic acid (PASP)

This biocompatible water soluble polymer was prepared from maleic anhydride and aqueous ammonia through a two- step procedure [18]. FTIR (KBr): ν (cm-1) = 538, 633, 708, 846, 940, 1109, 1236, 1351, 1392, 1549, 1712 (C = O carboxylate), 2880 (CH aliphatic), 2892 (CH aliphatic), 3100–3500 broad (OH). PASP acid was prepared as previously described by our group.^1^H-NMR (400 MHz, TMS, δ in ppm) 4.71 (CH in α segment), 4.57 (CH in β segment), 2.7 (CH_2_ β segment), Fig.SI.

#### Synthesis of PASP-mSiO2 Nanoparticles

Initially, an aqueous solution of PASP (pH 5.2) was subjected to lyophilization at -45^°^C. Subsequently, a mixture of the lyophilized PASP (100 mg), EDC.HCl (30 mg), NHS (30 mg), 5 mL of anhydrous DMSO and 15 mL of PBS (pH = 4.4) was sonicated for 20 min. To this solution, 300 mg of mSiO_2_-NH_2_ was added drop wise. The mixture was then stirred (400 rpm) in the dark for 2 days. The solid was separated by centrifugation (8000 rpm, 10 min). The yellowish white solid was thoroughly washed with DI (3 × 20 mL) and methanol (3 × 20 mL) and dried under reduced pressure. The solid was stored in the dark under argon at 4 ^°^C.

#### Synthesis of PASP-mSiO2-DTPA nanoparticles

DTPA dianhydride (30 mg) of the prepared mixture was added to a mixture of NHS (30 mg) and DCC (100 µL) previously dissolved in 5 mL of anhydrous DMSO. The mixture was then sonicated at 50 ^°^C for 3 h using an ultrasonic water bath. After cooling to room temperature, 20 mg of *m*SiO_2_-PASP was added and the mixture was magnetically stirred (400 rpm) in the dark for 48 h. The nanoparticles were collected through centrifugation and washed with DI (3 × 20 mL) and ethyl alcohol (3 × 20 mL). The yellowish white solid was then dried under reduced pressure and stored under argon.

#### ^99 m^Tc radiolabeling

Radiolabeling of mSiO_2_-DTPA nanoparticles and PASP-mSiO_2_-DTPA nanoparticles with ^99m^Tc was performed using SnCl_2_.2H_2_O as a reducing agent [19]. Briefly, 0.1 M HCl solution containing different amounts of SnCl_2_.2H_2_O (0.5–4 µg) was added to sealed evacuated vials each containing 1 mg of mSiO_2_-DTPA nanoparticles or PASP-mSiO_2_-DTPA nanoparticles dispersed in 1 mL of water. The radiolabeling reaction started with the addition of 0.5 mL ^99m^TcO_4_^−^ (37 MBq). The reaction mixture was incubated at ambient temperature (25 ± 5 ^º^C) for different time intervals (5–30 min) to determine the optimum reaction time. The radiochemical yield was estimated using thin layer chromatographic technique (TLC) utilizing silica gel- coated aluminum foil strips as a stationary phase. Two eluents were used; the first one was acetone for the determination of the free technetium (^99m^TcO_4_^**−**^) percentage. The second eluent was a mixture of water, ethanol and ammonium hydroxide (5: 2: 1 v/v/v) for the determination of reduced hydrolyzed ^99m^Tc and stannous colloid. All experiments were performed in triplicate and the radioactivity was estimated using a well type NaI (TI) gamma counter.

### In vitro stability study

The stability of technetium- 99 m labeled mSiO_2_-DTPA nanoparticles and PASP-mSiO_2_-DTPA nanoparticles was investigated in PBS and fetal bovine serum albumin (FBS). The experiments were performed by incubating 1 mL of aqueous dispersions of radiolabeled nanoparticles with 3 mL of PBS or FBS for 24 h at 37^º^C. At different time intervals (0.5, 1, 2, 4, 6, and 24 h) aliquots were taken out and the free technetium percentage was determined using TLC under the same conditions mentioned before.

### Hydroxyapatite microspheres binding assay

To determine the degree of binding of the synthesized nanoparticles to bone, a hydroxyapatite micro sphere binding assay was conducted. Initially, HA was prepared as described in the literature [20]. Dispersion of technetium radiolabeled mSiO_2_-DTPA-^99m^Tc nanoparticles and PASP-mSiO_2_-DTPA- ^99m^Tc nanoparticles (3.7 MBq in 1 mL PBS) were incubated with 5 mg HA for 2 h at 37 °C. The incubated sample was then separated by centrifugation at 500 rpm for 5 min; and the radioactivity of the collected supernatant was measured. This experiment was repeated three times. The relative binding affinity of the radiolabeled nanoparticles was calculated using the following equation:

$$\begin{aligned}&\:HA\:binding\:\left(\%\right)\cr&=\frac{(\text{t}\text{o}\text{t}\text{a}\text{l}\:\text{r}\text{a}\text{d}\text{i}\text{o}\text{a}\text{c}\text{t}\text{i}\text{v}\text{i}\text{t}\text{y}\:-\:\text{s}\text{u}\text{p}\text{e}\text{r}\text{n}\text{a}\text{t}\text{a}\text{n}\text{t}\:\text{r}\text{a}\text{d}\text{i}\text{o}\text{a}\text{c}\text{t}\text{i}\text{v}\text{i}\text{t}\text{y})}{total\:radioactivity}\:\times100\end{aligned}$$

### Biocompatibility study

The biocompatibility of both mSiO_2_-DTPA nanoparticles and PASP-mSiO_2_-DTPA nanoparticles was assessed using an MTT assay. The cytotoxicity was assessed against human foreskin fibroblast cells (Hs27) as representative of normal human cells. The impact of concentrations (0–200 µg /mL) of nanoparticles on cell viability was determined by incubating them with the cells for 48 h at 37 °C.

### Biodistribution study

Evaluation of the bone targeting efficiency of the synthesized silica nanoparticles was evaluated by studying the biodistribution profile of the ^99m^Tc radiolabeled nanoparticles in normal male Swiss Albino mice (25–30 g). Briefly, a 150 µL aqueous dispersion of ^99m^Tc radiolabeled mSiO_2_-DTPA nanoparticles or PASP-mSiO_2_-DTPA nanoparticles containing 100 µg nanoparticles and equivalent to 500 µCi was injected intravenously into the tail vein of each normal mouse. At various time intervals (0.5, 1, 2, 4 and 6 h post- injection, with 5 mice for each time point), the mice were anesthetized and sacrificed humanely by cervical dislocation. Different mice organs were dissected and washed twice with saline and weighed. Samples of fresh blood, bone and muscle were collected and weighed. Blood, bone and muscles were assumed to be 7, 10 and 40% of the total body weight, respectively [21]. The radioactivity of each organ was measured using a gamma counter and expressed as a percentage of the injected activity per gram organ or body fluid (% IA/gram organ or body fluid). All results are presented as the mean value ± standard deviation (SD).

### Statistical analysis

Statistical analysis of the data was performed using One-way ANOVA. The differences were considered statistically significant at *p* < 0.05.

## Results and discussion

### Synthesis and characterization

**Fig. 1:**
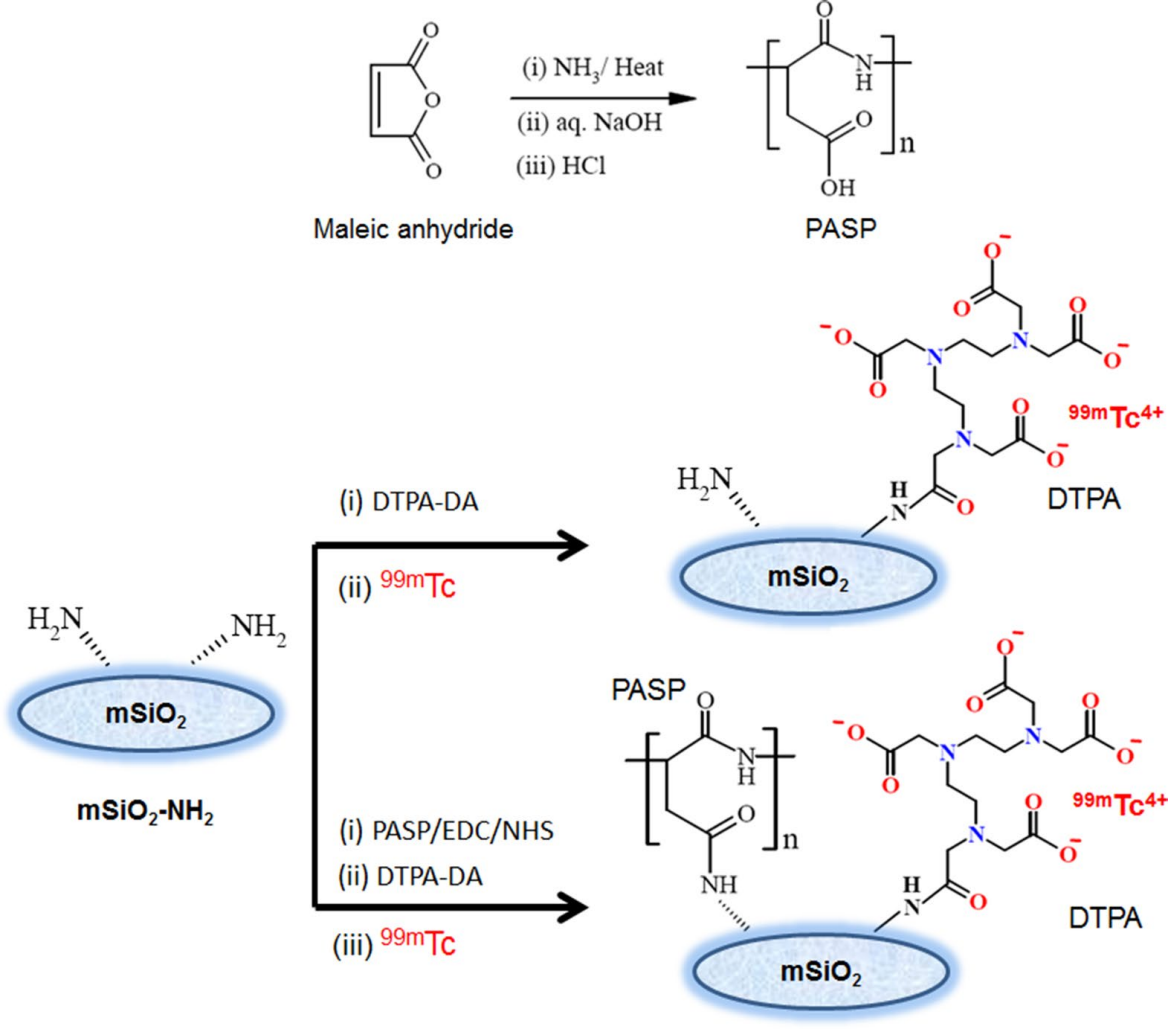
Schematic representation for preparation of PASP, mSiO_2_-DTPA nanoparticles, and PASP-mSiO_2_-DTPA nanoparticles

For sensitive and non-invasive SPECT bone-imaging, the moiety to be radiolabeled should interact exclusively with bone without toxic degradable products. Mesoporous silica nanoparticles (MSNs) and polyamino acids such as polyaspartic acid (PASP) show great potential in this regard. The aim of this work was to develop an effective bone-targeting probe based on the biocompatibility and bone-targeting features of both mesoporous silica nanoparticles (mSiO_2_-NH_2_) and polyaspartic acid (PASP). First, a facile one-pot method was used to prepare amino-functionalized mesoporous silica nanoparticles (mSiO_2_-NH_2_) byco-condensation of silica precursor TEOS with an amino silane coupling agent (APTS) in the presence of CTAB as a shape directing agent [16]. Compared to the classical two-step method, the co-condensation method is more facile and proved to ensure a more homogeneous distribution of amino groups on the surface of the silica nanoparticles [22]. When further covalent binding of active molecules (e.g. drugs or molecules for active targeting, etc.) to the surface of silica is intended, the homogeneous distribution of anchoring amino groups is of utmost importance. Amino-functionalized silica (mSiO_2_-NH_2_) nanoparticles were then conjugated to diethylene- triaminepentaacetic dianhydride (DTPA-DA) to produce mSiO_2_-DTPA nanoparticles (Fig. 1) [23]. In another probe, the biocompatible PASP was synthesized and subsequently anchored onto the amino groups present at the surface of mSiO_2_-NH_2_ nanoparticles to produce SiO_2_-PASP nanoparticles (Fig. 1). Subsequently, DTPA was anchored to the unreacted amino groups to produce PASP-mSiO_2_-DTPA nanoparticles. Both types of nanoparticles were successfully radiolabeled with Technetium-99m to produce mSiO_2_-DTPA-^99m^Tc nanoparticles and PASP-mSiO_2_-DTPA-^99m^Tc nanoparticles suitable for in vivo SPECT bone imaging. The carboxylic groups were activated to NHS-ester groups via simple aqueous carbodiimide chemistry in all coupling reactions.

**Fig. 2:**
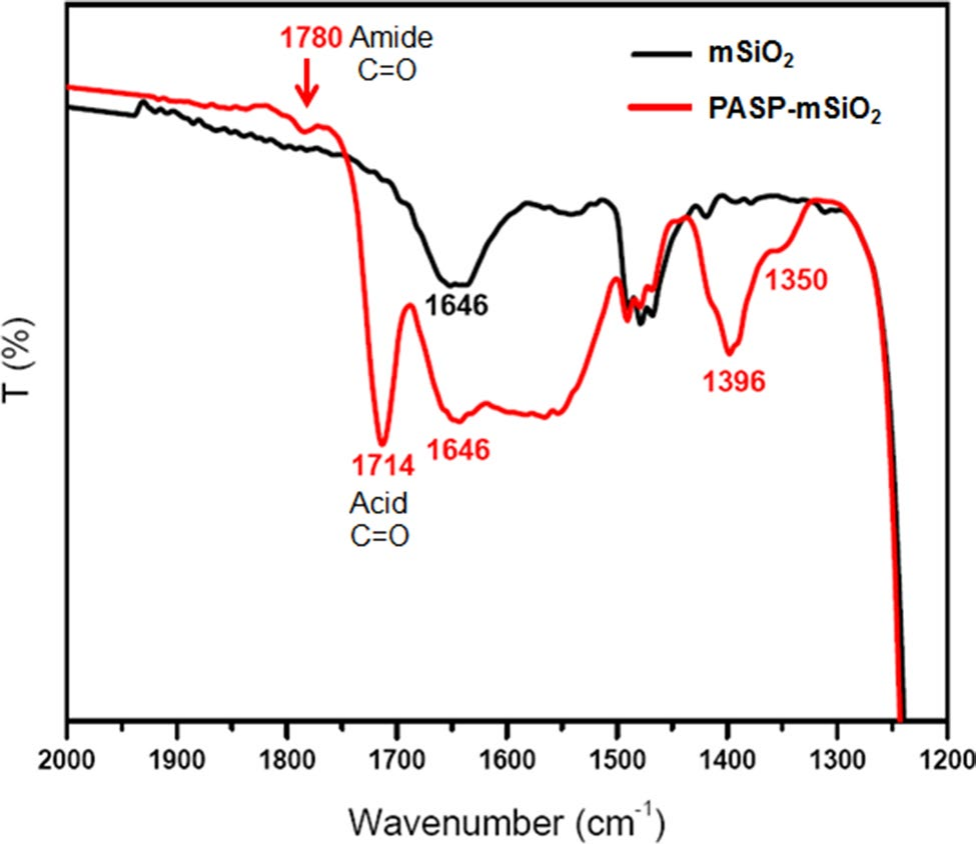
ATR-FTIR spectra, **a**, UV-Visible spectra, **b** and Thermal gravimetric analysis (TGA), **c** of mSiO_2_ nanoparticles and PASP-mSiO_2_ nanoparticles

The prepared nanoparticles were characterized by FTIR, TEM, FE-SEM, EDX, XRD, and TGA. The FTIR spectra of mSiO_2_-NH_2_ nanoparticles and PASP-mSiO_2_-NH_2_ nanoparticles matched well with the data previously reported for mSiO_2_-NH_2_ [22] and PASP [18]. The broad peak at 3000–3600 cm^− 1^ assigned to the Si-OH group of silica nanoparticles or adsorbed water molecules [24a]. The most characteristic region in the FTIR spectra (1200 cm^− 1^ -2000 cm^− 1^) of mSiO_2_-NH_2_ nanoparticles and PASP-mSiO_2_-NH_2_ nanoparticles is shown in Fig. 2a. PASP-coated silica showed two bands at 1714 cm^− 1^ and 1780 cm^− 1^ corresponding to stretching vibrations of the carboxylic carbonyl group and amide carbonyl group, respectively [24a]. This indicates that some carboxylic groups of PASP linked to the amino groups at the surface of mSiO_2_-NH_2_ forming an amide linkage, while other groups remain unbound to silica. Vibration peaks in the range of 400 cm^− 1^ − 1400 cm^− 1^ are characteristic for symmetric stretching and bending of the Si-O-Si bond. The bending mode of the amino groups is observed at 1646 cm^− 1^. The C-H stretching modes of the methylene groups of the aminopropyl chain and PASP chains give rise to signals in the 2848–2920 cm^− 1^ range.

**Fig. 3:**
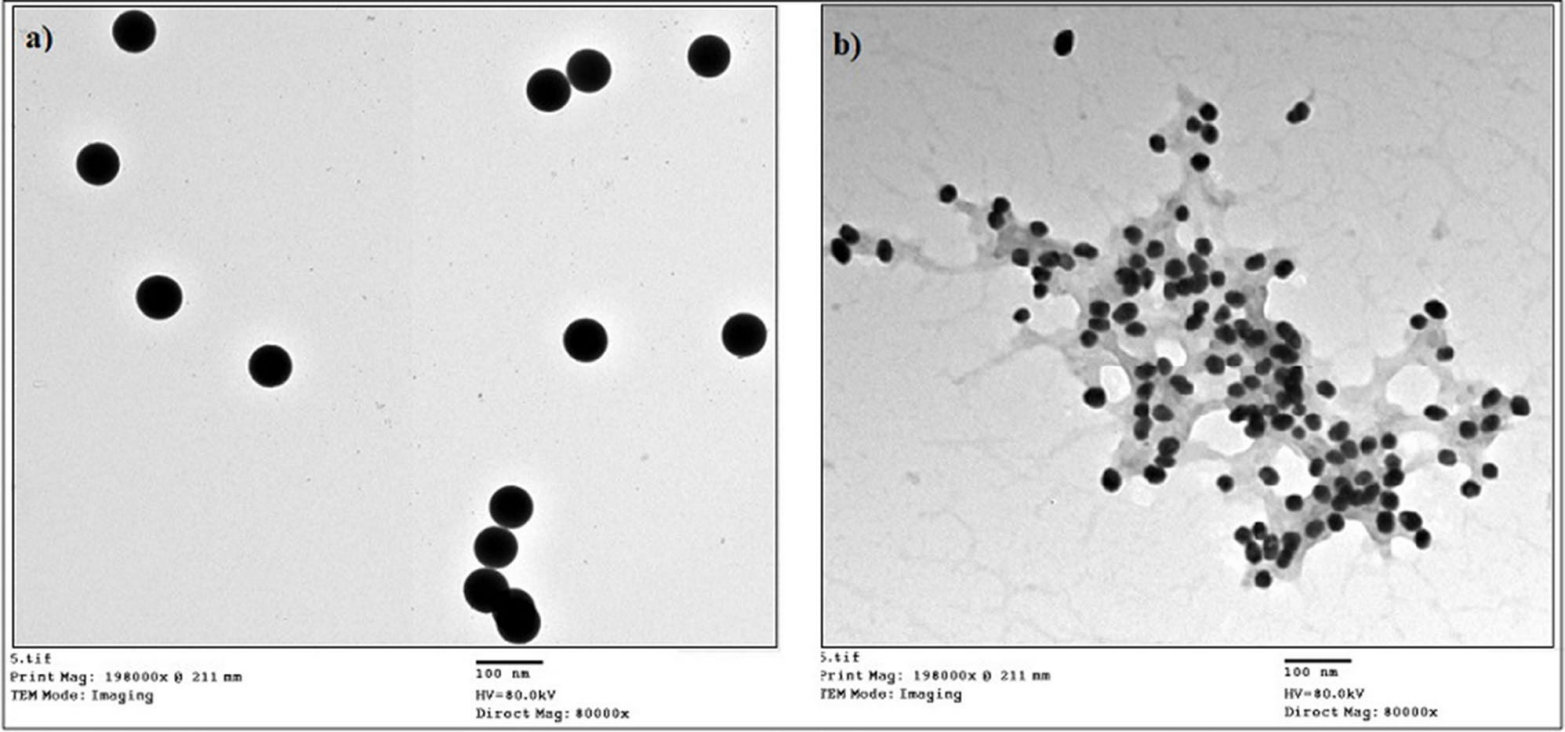
TEM images of mSiO_2_ nanoparticles, **a**, and PASP-mSiO_2_ nanoparticles, **b**

**Fig. 4:**
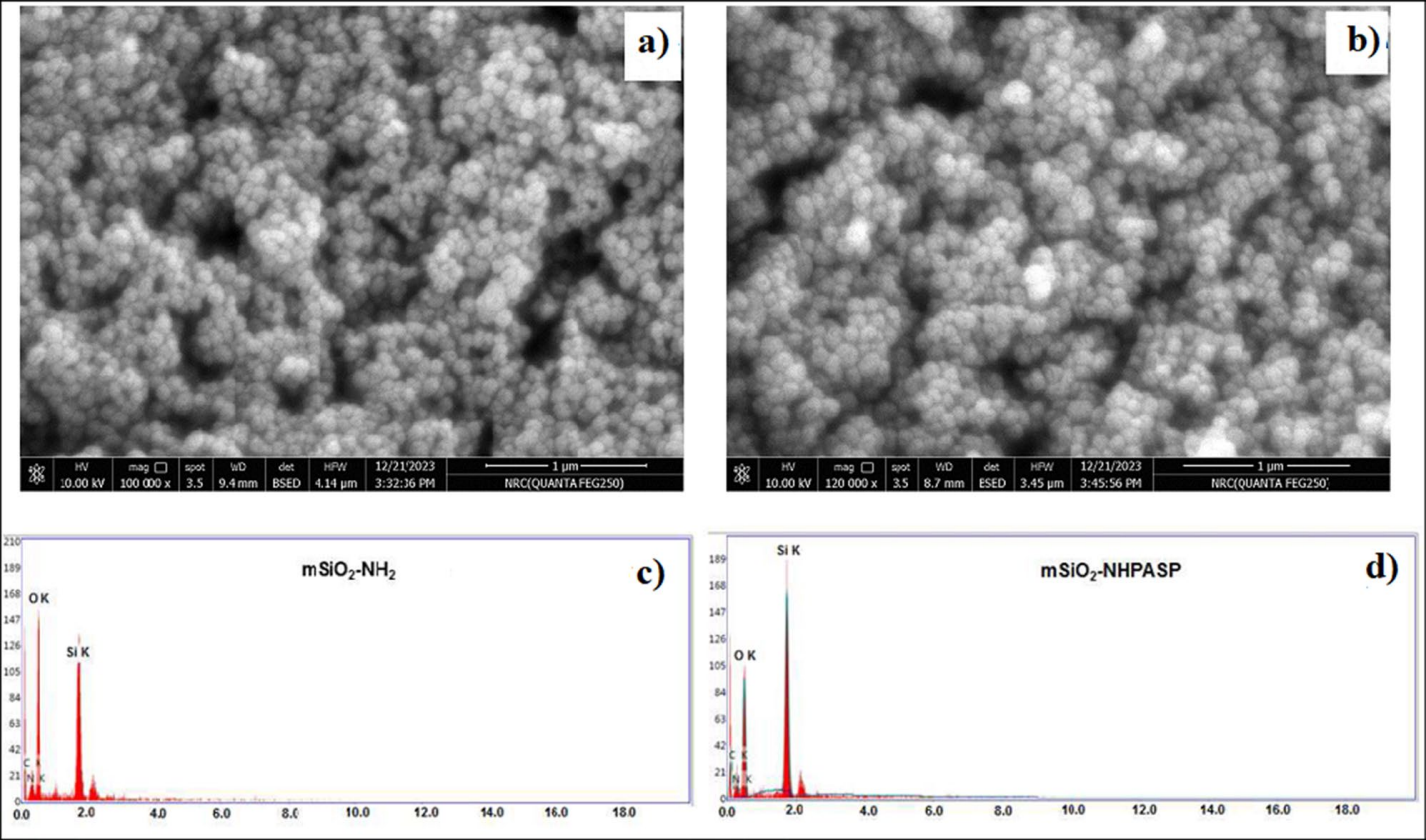
FE-SEM images of mSiO_2_ nanoparticles, **a**, and PASP-mSiO_2_ nanoparticles, **b**, EDX mapping, **d** of mSiO_2_ nanoparticles, **c**, and PASP-mSiO_2_ nanoparticles

**Fig. 5:**
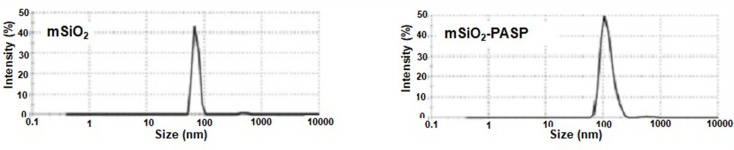
DLS images of mSiO_2_ nanoparticles, a, and mSiO_2_- PASP nanoparticles

UV-Vis spectra showed that all prepared nanoparticles are inactive in the visible region of spectra (400–700 nm). However, the absorption profiles in the UV region (200–400 nm) (Fig. 2b). Silica is optically inactive in this region, while conjugation to DTPA or PASP resulted in an increase in absorbance in the UV region. This observation gives rise to believe in particular changes of the chemical bonding schemes at the surface of silica nanoparticles. Thermal gravimetric analysis (TGA) of PASP, mSiO_2_ nanoparticles and PASP-mSiO_2_ nanoparticles was carried out under a nitrogen atmosphere to avoid any expected thermal degradation (Fig. 2c). In contrast to the high thermal stability of mSiO_2_ nanoparticles, pure PASP undergoes a rapid thermal degradation after 200 ^°^C. Considering these results and comparing the thermograph of mSiO_2_ nanoparticles and PASP-mSiO_2_ nanoparticles, it can be concluded that the PASP layer represents about 10% of the weight of PASP-mSiO_2_ nanoparticles. X- ray diffraction patterns of mSiO_2_ and PASP-mSiO_2_ are shown in Fig. SII. MSiO_2_ showed some degree of crystallinity in the range of 2θ = 10–30 [23]. Interestingly, PASP coat showed a higher degree of crystallinity as indicated by sharp peaks from 2θ = 20–30 [18]. TEM images (Fig. 3a and b) indicate that both mSiO_2_-NH_2_ and mSiO_2_-PASP nanoparticles are monodispersed and have a spherical morphology. Coating with PASP did not change either size (~ 58 ± 6 nm) or shape of mSiO_2_-NH_2_ (size was ascertained by measuring 50 particles in each material). Coating with PASP resulted in the formation of a polymer corona around mSiO2-NH2. The formation of a corona structure is well known when inorganic nanoparticles are coated with a hydrophilic polymer [24b]. FE-SEM images of both mSiO_2_ nanoparticles, PASP-mSiO_2_ nanoparticles showed monodispersed spherical nanoparticles of a smooth surface (Fig. 4a and b). The mean diameters of mSiO_2_ nanoparticles, PASP-mSiO_2_ nanoparticles were found to be around 80 and 95 nm, respectively. Energy dispersive X-ray (EDX) analysis of mSiO_2_ nanoparticles and PASP-mSiO_2_ nanoparticles (Fig. 4c and d; Table 1) showed an increase in the percent content of carbon and oxygen while there was a decrease in the percent content of silicon after anchoring with PASP. As shown in Fig. 5, DLS analysis revealed that conjugation of PASP increased the hydrodynamic size of silica nanoparticles from 95 ± 7 nm (PDI 0.412) to 120 ± 9 nm (PDI 0.521). This increase can be attributed to the outermost layer of PASP and the enhanced water absorbability of PASP-mSiO_2_ nanoparticles [24a]. The zeta potentials of mSiO_2_ NPs and PASP-mSiO_2_ NPs in their aqueous dispersions were + 18.22 and + 10.11, respectively. The positively charged surface of amino modified silica is due to the amino groups on the surface of the NPs. The surface positive charge was decreased after substitution of some of the surface amino groups with anionic polyaspartate.

**Table 1:** Data of EDX analysis of mSiO_2_-NH_2_ nanoparticles and PASP-mSiO_2_ nanoparticles

Element	% C	% *N*	% O	% Si
mSiO_2_-NH_2_	15	10.92	51.29	14.16
PASP-mSiO_2_	20.48	11.81	53.12	12.59

X-ray diffraction patterns of mSiO_2_ nanoparticles and PASP-mSiO_2_ nanoparticles are shown in Fig. SI. In addition to the peaks characteristic of silica nanoparticles, the anchoring of PASP resulted in the appearance of new peaks at higher 2theta values.

### ^**99 m**^**Tc radiolabeling and** in vitro **Stability study**

The radiolabeling of mSiO_2_ nanoparticles and PASP-mSiO_2_ nanoparticles with ^99m^Tc was conducted using a DTPA chelation procedure (Fig. 1). MSiO_2_-DTPA nanoparticles and PASP-mSiO_2_-DTPA nanoparticles were radiolabeled with ^99m^Tc in high yield as indicated by TLC analysis. This analysis technique is a preferable method for radiochemical yield estimation because it is simple, fast and reliable [23]. According to the TLC conditions applied in this study, the radiolabeled nanoparticles remain at the baseline with the colloid (R_f_ = 0) when using acetone as an eluent while the free technetium (^99m^TcO_4_^−^) moves with the solvent front (R_f_ =0.9-1). When using the previously mentioned mixture as an eluent, the colloid remains at the baseline while silica nanoparticles and the free technetium move with the eluent (R_f_ =0.6–0.7 and R_f_ = 0.9-1, respectively). The maximum radiolabeling yield achieved for mSiO_2_-DTPA nanoparticles and PASP-mSiO_2_-DTPA nanoparticles was (97 ± 0.5% and 92 ± 0.5%, respectively) using 1 µg of SnCl_2_.2H_2_O. The radiochemical yield was determined after different time intervals and the results revealed that the optimum reaction time for obtaining maximum yield was 15 min. High in vitro stability (ranging from 96 to 98% stable radiolabeled nanoparticles) was observed for both types of radiolabeled silica nanoparticles in PBS and FBS up to 24 h. This convenient stability has encouraged further biodistribution studies.

### Hydroxyapatite binding affinity

**Fig. 6:**
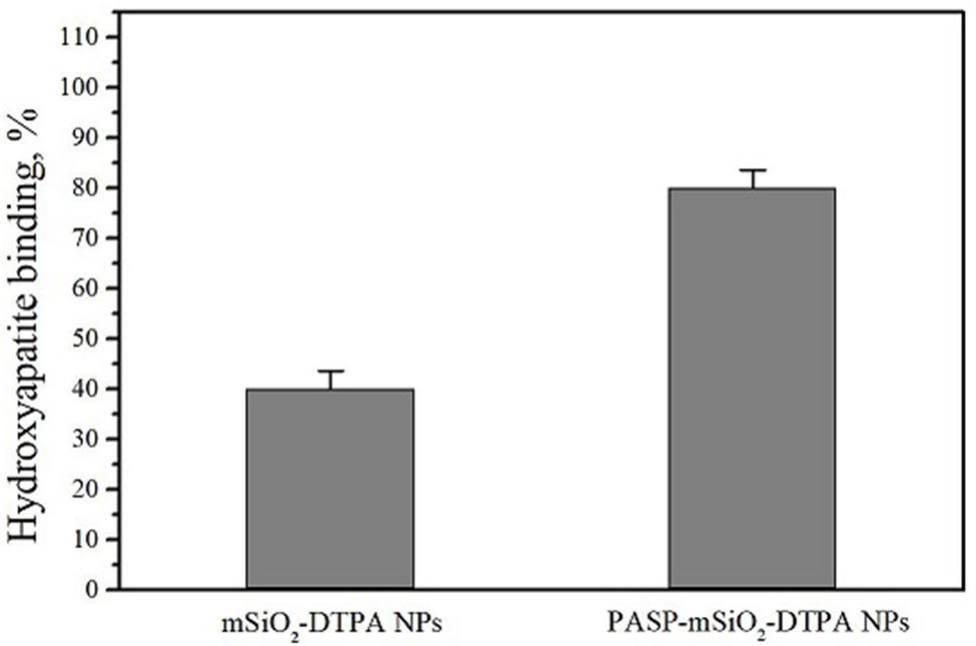
Hydroxyapatite binding affinity of mSiO_2_ nanoparticles, a, and mSiO_2_- PASP nanoparticles

The human body^’^s bone are rich in hydroxyapatite (HA) so enhancement of nanoparticles bone targeting can be achieved by increasing HA binding affinity. In this study, in vitro evaluation of the effect of poly aspartic acid conjugation to silica nanoparticles on bone affinity was performed by determining the relative binding of the radiolabeled nanoparticles to HA microsphere. The results shown in Fig. 6 revealed higher HA affinity percent of PASP-mSiO_2_-DTPA nanoparticles compared to mSiO_2_-DTPA nanoparticles (80 ± 3% and 40 ± 3.5%, respectively) confirming the potential role of poly aspartic acid conjugation in enhancing the silica nanoparticles HA affinity and accumulation in the bone [11].

### Biocompatibility and biodistribution studies

**Fig. 7:**
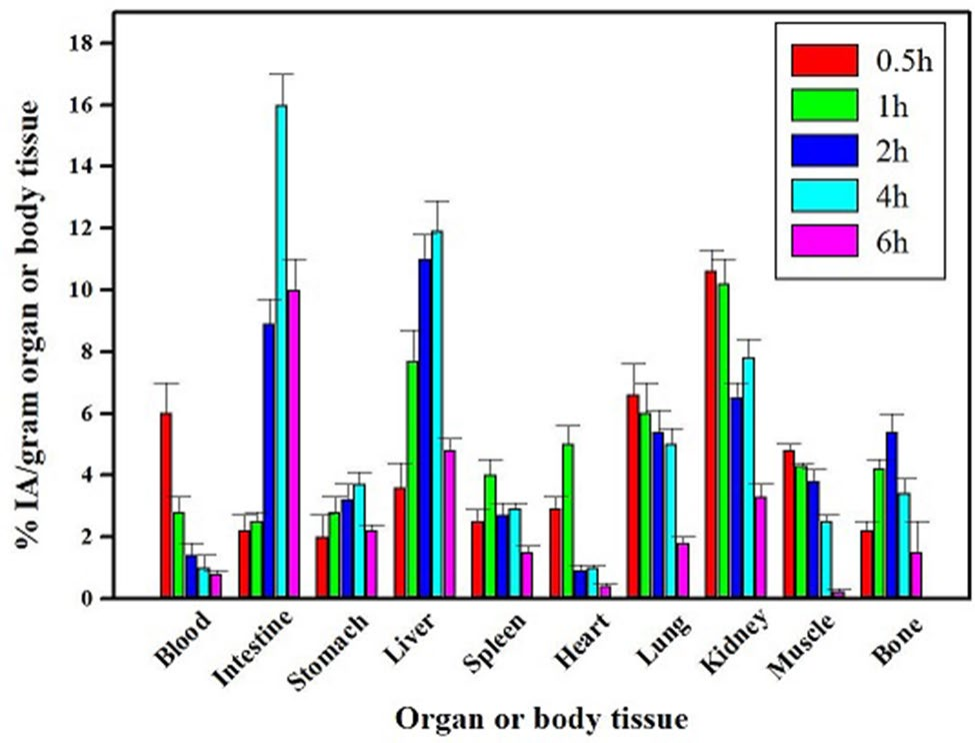
Biodistribution studies results of SiO_2_-DTPA-^99m^Tc after IV injection in mice, (*n* = 5/time point); IA for injected activity

**Fig. 8:**
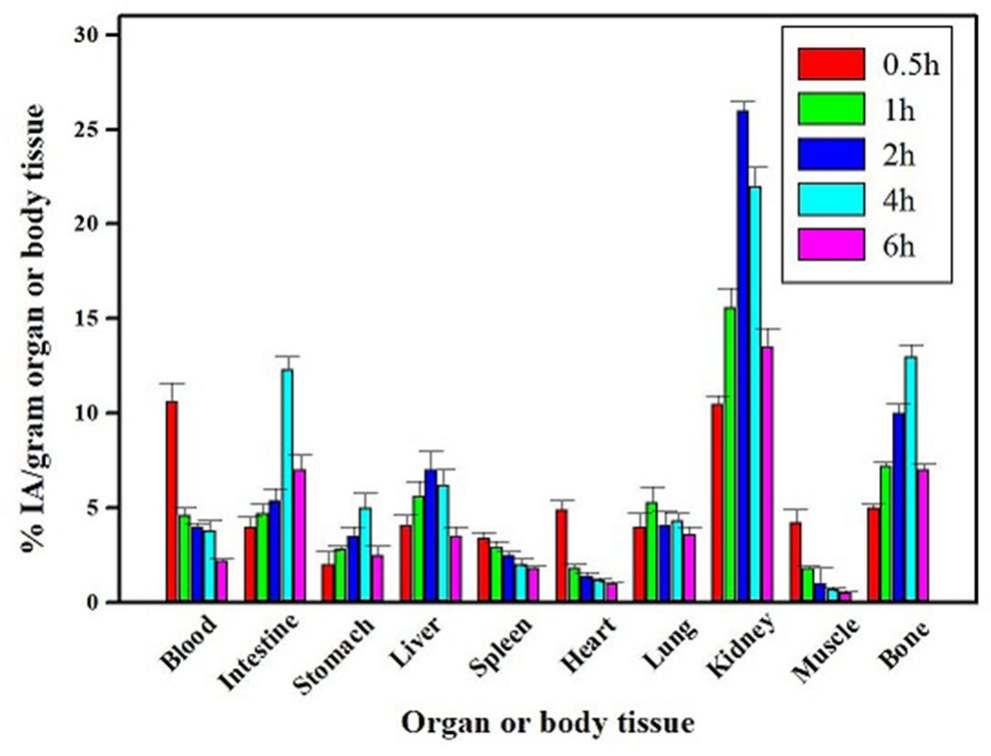
Biodistribution studies results of PASP-SiO_2_-DTPA-^99m^Tc after IV injection in mice, (*n* = 5/time point); IA for injected activity

**Fig. 9:**
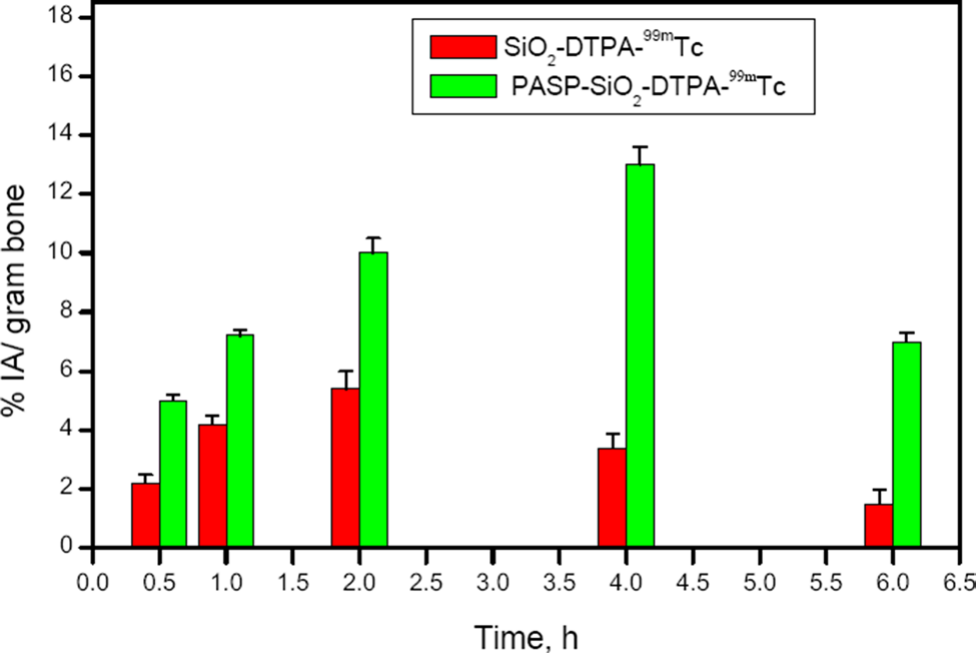
Bone uptake of SiO_2_-DTPA-^99m^Tc and PASP-SiO_2_-DTPA-^99m^Tc, (*n* = 5/time point), *statistically significant difference (*p* < 0.05); IA for injected activity

The biocompatibility of SiO_2_-DTPA nanoparticles and PASP-SiO_2_-DTPA nanoparticles was studied on a human fibroblast cell line. The MTT assay results revealed that incubation of the cells with the prepared nanoparticles at a concentration of up to 200 µg/ mL for 48 h induced undetectable cell toxicity (cell viability about 96 ± 0.5%). The biocompatibility of the prepared nanoparticles and the high in vitro stability of the radiolabeled nanoparticles encouraged performing a comparative biodistribution study. Determination of the radioactivity percentage in different organs of the experimental normal mice post intravenous injection of the ^99m^Tc labeled nanoparticles clarifies different data as shown in Figs. 7 and 8. A higher radioactivity level was detected in the kidney after injection of PASP-SiO_2_-DTPA-^99m^Tc nanoparticles compared to SiO_2_-DTPA-^99m^Tc nanoparticles (26 ± 0.5% IA/gram at 2 h post injection, 10.2 ± 0.8% IA/gram 1 h post injection, respectively). This may be attributed to the enhancement of hydrophilic properties of the silica nanoparticles via anchoring with polyaspartic acid which matches with the reported renal excretion of different radiolabeled oligo-aspartic acid [15]. ^99m^Tc-MDP, the clinically used bone imaging radiopharmaceutical also undergoes urinary excretion [25]. On the other hand, the results revealed the higher radioactivity level of SiO_2_-DTPA-^99m^Tc nanoparticles in the liver and intestine (11.9 ± 1 and 16 ± 1% IA/gram 4 h post injection, respectively) compared to that of PASP-SiO_2_-DTPA-^99m^Tc nanoparticles (6.2 ± 0.8% and 12.3 ± 0.7% IA/gram 4 h post injection, respectively). This indicates the hepatobiliary excretion of (SiO_2_-DTPA-^99m^Tc) nanoparticles which is consistent with the behavior of the previously studied bone targeting silica nanoparticles [26]. As shown in Fig. 9 SiO_2_-DTPA-^99m^Tc nanoparticles showed the maximum bone uptake (C _max_ equal to 5.4 ± 0.4% IA/gram) at t _max_ = 2 h post injection. A higher radioactivity accumulation level in the bone was detected after intravenous injection of PASP-SiO_2_-DTPA-^99m^Tc nanoparticles as shown in Fig. 8, C _max_ equal to 13 ± 0.6% IA/gram at t _max_= 4 h post injection. Figure 7 revealed the significant enhancement of radioactivity bone uptake (*p* < 0.05) after injection of PASP-SiO2-DTPA-^99m^Tc at different time points attributed to the high affinity of poly aspartic acid for hydroxyapatite of the bone [2, 27]. These results are consistent with the high bone accumulation level of ^67^Ga-DOTA-(Asp) _8_ (12.56 ± 3.09% IA/gram at 1 h post injection) which has been investigated previously as a PET bone imaging probe [15]. Interestingly, the bone uptake level of the injected synthesized SiO_2_-DTPA-^99m^Tc and PASP-SiO_2_-DTPA-^99m^Tc nanoparticles is higher than the previously reported bone uptake level of the injected ^99m^Tc-MDP (2.66% ID/g) [28]. These results encourage further studies of these designed ^99m^Tc silica nanoparticles as an alternative to traditional bisphosphonate radiopharmaceuticals. The in vivo stability of SiO_2_-DTPA-^99m^Tc and PASP-SiO_2_-DTPA-^99m^Tc nanoparticles was confirmed with the low radioactivity level detected in the thyroid gland (1.5 ± 0.3% and 2 ± 0.4% IA/gram, respectively) and stomach (3.2 ± 0.5% IA/gram and 3.5 ± 0.4% IA/gram, respectively) related to the free ^99m^Tc radioisotope due to the instability of the synthesized radiopharmaceutical [30]. Additionally, the radioactivity detected in the bone also reveals the in vivo stability of the radiolabeled silica nanoparticles because ^99m^Tc radionuclide does not accumulate in the bone in the free form [29].

## Conclusions

In this work, polyaspartic acid coated mesoporous silica (PASP-mSiO_2_) nanoparticles were synthesized by anchoring polyaspartic acid onto amino-functionalized silica nanoparticles via simple aqueous carbodiimide chemistry. The prepared nanoparticles exhibited monodispersibility, high physiological stability and biocompatibility. DTPA was covalently attached to the amino groups on the surface of the prepared nanoparticles allowing for effective labeling with the radiotracer, Technetium-99m. A biodistribution study revealed the highly selective accumulation of PASP-mSiO_2_ nanoparticles in bone, compared to uncoated silica nanoparticles. Due to their non-invasive and high bone-targeting features, the prepared ^99m^Tc-labeled PASP-mSiO_2_ nanoparticles have high potential for safe and sensitive SPECT imaging of bone. Further studies are needed to evaluate the prepared nanoparticles as probes for targeted radionuclide palliation of metastatic bone pain.

## Electronic supplementary material

Below is the link to the electronic supplementary material.

Supplementary Material 1

Supplementary Material 2

## Data Availability

Data is provided within the manuscript and supplementary information files.

## References

[1] Crișan G, Moldovean-Cioroianu NS, Timaru DG, Andrieș G, Căinap C, et al. Radiopharmaceuticals for PET and SPECT imaging: A literature review over the last decade. Int J Mol Sci. 2022;30:5023–61. 10.3390/ijms23095023 PMC9103893 35563414

[2] Uehara T, Kiyota S, Ishii D, Ogawa K, Akizawa H, et al. Mononuclear Tc-99m-chelate-conjugated oligo-aspartic acid as a new bone imaging agent. J Nucl Med. 2006;47:520.16513622

[3] Mushtaq S, Bibi A, Park JE, Jeon J. Recent progress in technetium-99m-labeled nanoparticles for molecular imaging and cancer therapy. Nanomater. 2021;11:3022–49. 10.3390/nano11113022 PMC8618883 34835786

[4] Gisbert-Garzarán M, Manzano M, Vallet-Regí M. Mesoporous silica nanoparticles for the treatment of complex bone diseases: bone cancer, bone infection and osteoporosis. Pharm. 2020;12:83–110. 10.3390/pharmaceutics12010083 PMC7022913 31968690

[5] Aguilera-Correa JJ, Gisbert-Garzarán M, Mediero A. Antibiotic delivery from bone-targeted mesoporous silica nanoparticles for the treatment of osteomyelitis caused by methicillin-resistant Staphylococcus aureus. Acta Biomater. 2022;154:608–25. 36341887 10.1016/j.actbio.2022.10.039

[6] Nie B, Huo S, Qu X, Guo J, Liu X, et al. Bone infection site targeting nanoparticle-antibiotics delivery vehicle to enhance treatment efficacy of orthopedic implant related infection. Bioact Mater. 2022;12:134–48. 10.1016/j.bioactmat.2022.02.003 PMC8958424 35386313

[7] Jia Y, Zhang P, Sun Y, Kang Q, Xu J, et al. Regeneration of large bone defects using mesoporous silica coated magnetic nanoparticles during distraction osteogenesis. Nanomed Nanotechnol Biol Med. 2019;21:102040–55. 10.1016/j.nano.2019.102040 31228602

[8] Xu c, Xiao L, Cao Y, Sun w. Mesoporous silica rods with cone shaped pores modulate inflammation and deliver BMP-2 for bone regeneration. Nano Res. 2020;13:2323–31.

[9] Pasqua L, Napoli IE, Santo M, Greco M, Catizzone E. Mesoporous silica-based hybrid materials for bone-specific drug delivery. Nanoscale Adv. 2019;1:3269–78. 36133588 10.1039/c9na00249a PMC9417532

[10] Adelnia H, Tran HDN, Little PJ, Blakey I, Ta HT. Poly (aspartic acid) in biomedical applications: from polymerization, modification, properties, degradation, and biocompatibility to applications. CS Biomater Sci Eng. 2021;7:2083–105. 10.1021/acsbiomaterials.1c00150 33797239

[11] Kasugai S. Selective drug delivery system to bone: small peptide (Asp)_6_ conjugation. J Bone Min Res. 2000;15:936–43. 10.1359/jbmr.2000.15.5.936 10804024

[12] Wang D, Miller S, Sima M, Kopecková P, Kopecek J. Synthesis and evaluation of water-soluble polymeric bone-targeted drug delivery systems. Bioconjug Chem. 2003;14:853–9. 13129387 10.1021/bc034090j

[13] Ouyang L, Pan J, Zhang Y, Guo L. Synthesis of second- and third-generation asp oligopeptide conjugated dendrimers for bone-targeting drug delivery. Synth Commun. 2009;39:4039–52.

[14] Jiang T, Yu X, Carbone EJ, Nelson C, Kan HM, et al. Poly aspartic acid peptide-linked PLGA based nanoscale particles: potential for bone-targeting drug delivery applications. Int J Pharm. 2014;475:547–57. 25194353 10.1016/j.ijpharm.2014.08.067

[15] Ogawa K, Ishizaki A, Takai K, Kitamura Y, Kiwada T, et al. Development of novel radiogallium-labeled bone imaging agents using oligoaspartic acid peptides as carriers. PLoS ONE. 2013;8:e84335. 24391942 10.1371/journal.pone.0084335 PMC3877283

[16] Wada A, Tamaru S, Ikeda M, Hamachi I. MCM- enzyme-supramolecular hydrogel hybrid as a fluorescence sensing material for polyanions of biological significance. J Am Chem Soc. 2009;131:5321–30. 19351208 10.1021/ja900500j

[17] Ardestani MS, Arabzadeh AJ, Heidari Z. Novel and facile methods for the synthesis of DTPA-mono-amide: a new completely revised strategy in radiopharmaceutical chemistry. J Radioanal Nucl Chem. 2010;283:447–55.

[18] Niţà LE, Chiriac AP, Popescu CM, Neamţu I, Alecu L. Possibilities for Poly (aspartic acid) Preparation as biodegradable compound. J Optoelectron Adv Mater. 2006;8:663–6.

[19] Amin AM, Abou Zid K, Bayoumi NA, Abd EL-hamid M. Organic synthesis and biological evaluation of novel ‘‘3 + 1’’ mixed ligands of technetium-99m Gabapentin as receptor imaging agents. J Radioanal Nucl Chem. 2010;283:55–62.

[20] Zhang CM, Yang J, Quan ZW, Yang PP, Li CX, et al. Hydroxyapatite nano and microcrystals with multiform morphologies: controllable synthesis and luminescence properties. Cryst Growth. 2009;9:2725–33.

[21] Bayoumi NA, Emam AN. ^99m^Tc radiolabeling of polyethylenimine capped carbon Dots for tumor targeting: synthesis, characterization and biodistribution. Int J Rad Biol. 2021;97:977–85. 33900891 10.1080/09553002.2021.1919781

[22] Estevão BM, Miletto I, Hioka N, Marchese L, Gianotti E. Mesoporous silica nanoparticles functionalized with amino groups for biomedical applications. Chem Open. 2021;10:1251–9. 10.1002/open.202100227 PMC8671895 34907672

[23] Barros ALB, Ferraz KSO, Dantas TCS. Synthesis, characterization, and biodistribution studies of ^99m^Tc-labeled SBA-16 mesoporous silica nanoparticles. Mater Sci Eng C. 2015;56:181–8. 10.1016/j.msec.2015.06.030 26249579

[24] Hakeem A, Zahid F, Zhan G, Yi P, Yang H, Darwish W, Abdoon A, Shata M, Elmansy M, et al. editors. Vincristine-loaded polymeric corona around gold nanorods for combination (chemo-photothermal) therapy of oral squamous carcinoma. React. Funct. Polym. 2020; 151: 104575.

[25] Truluck CA. Nuclear medicine technology: inflammation and infection imaging. J Radiological Nurs. 2007;26:77–85.

[26] Ren H, Chen S, Jin Y, Zhang C, Yang X, et al. Traceable and bone-targeted nanoassembly based on defectrelated luminescent mesoporous silica for enhanced osteogenic differentiation. J Mater Chem B. 2017;8:1585–93. 10.1039/c6tb02552h 32263930

[27] Yokogawa K, Miya K, Sekido T, Higashi Y, Nomura M. Selective delivery of estradiol to bone by aspartic acid oligopeptide and its effects on ovariectomized mice. Endocrinology. 2001;142:1228–33. 11181539 10.1210/endo.142.3.8024

[28] Mandiwana V, Kalombo L, Grobler A, Zeevaart JR. ^99m^Tc-MDP as an imaging tool to evaluate the *in vivo* biodistribution of solid lipid nanoparticles. Appl Radiat Isot. 2018;141:51–6. 30170270 10.1016/j.apradiso.2018.08.015

[29] Schwartz Z, Shani J, Soskolne WA, Touma H, Amir D. Uptake and biodistribution of technetium-99m-MD^32^P during rat tibial bone repair. J Nucl Med. 1993;34(1):104–8. 8418249

[30] Mushtaq S, Bibi A, Park JE, Jeon J. Recent progress in Technetium-99m-Labeled nanoparticles for molecular imaging and Cancer therapy nanomaterials 2021; 11: 3022. 10.3390/nano11113022 PMC8618883 34835786

