# Supplementary figures and images for: Synthesis of 99mTc-labeled polyaspartic acid/silica nanoassembly as a potential probe for bone imaging
Source: BMC Chem. 2025 May 24;19(1):142. doi: 10.1186/s13065-025-01508-z (PMC12102942; doi:10.1186/s13065-025-01508-z)

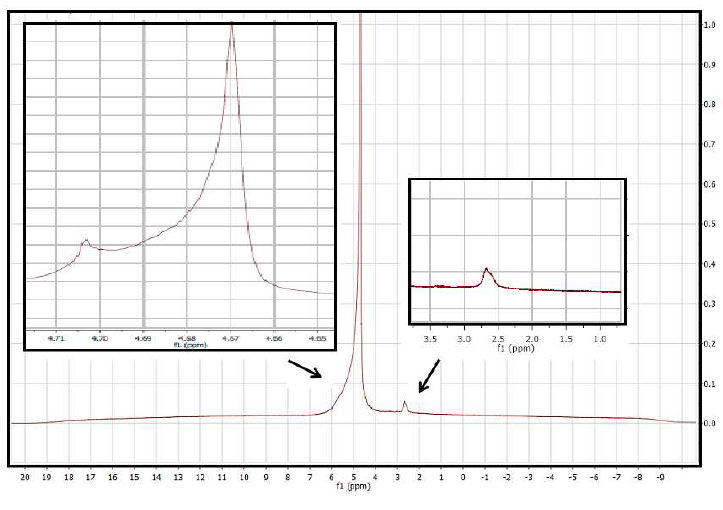

Supplement: Supplementary file 1 — Supplementary Material 1 [file 13065_2025_1508_MOESM1_ESM.tif]

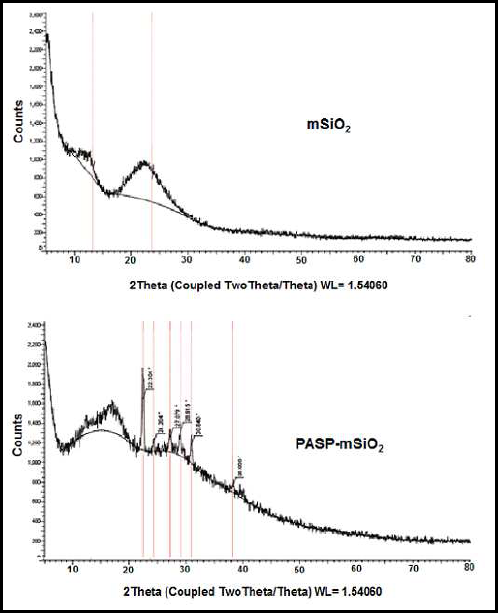

Supplement: Supplementary file 2 — Supplementary Material 2 [file 13065_2025_1508_MOESM2_ESM.tif]
